# Usage Metrics of Web-Based Interventions Evaluated in Randomized Controlled Trials: Systematic Review

**DOI:** 10.2196/15474

**Published:** 2020-04-16

**Authors:** Elena Koneska, Duncan Appelbe, Paula R Williamson, Susanna Dodd

**Affiliations:** 1 Department of Biostatistics University of Liverpool Liverpool United Kingdom; 2 Oxford Trauma, Nuffield Department of Orthopaedics Rheumatology & Musculoskeletal Sciences University of Oxford Oxford United Kingdom

**Keywords:** internet, web-based interventions, randomized controlled trial, web usage data, systematic review

## Abstract

**Background:**

The evaluation of web-based interventions (defined as an intervention that can be downloaded or accessed on the internet through a web browser) in randomized controlled trials (RCTs) has increased over the past two decades. Little is known about how participants’ use of the intervention is measured, reported, and analyzed in these studies.

**Objective:**

This study aimed to review the evaluation of web-based interventions in RCTs, assessing study characteristics and the methods used to record, and adjust for, intervention usage.

**Methods:**

A systematic review of the literature was undertaken to identify all published reports of RCTs that involved a web-based intervention. A random sample of 100 published trials was selected for detailed data extraction. Information on trial characteristics was extracted, including whether web usage data were recorded, and if so, the methods used to gather these data and whether these data were used to inform efficacy analyses.

**Results:**

A PubMed search identified 812 trials of web-based interventions published up to the end of 2017 and demonstrated a growing trend over time. Of the 100 studies reviewed, 90 studies collected web usage data, but more than half (49/90, 54%) of these studies did not state the method used for recording web usage. Only four studies attempted to check on the reliability of their web usage data collection methods. A total of 39% (35/90) studies reported patterns or levels of web intervention use, of which 21% (19/90) studies adjusted for intervention use in their outcome analysis, but only two of these used appropriate statistical methods.

**Conclusions:**

Trialists frequently report a measure of web-based intervention usage but do not always report the collection method or provide enough detail on their analysis of web usage. Appropriate statistical methods to account for intervention use are rarely used and are not well reported even in the very few trials in which they are used. The number of trialists who attempt to check on the reliability of their web usage collection methods is extremely low.

## Introduction

### Randomized Controlled Trials

A randomized controlled trial (RCT) is used to assess the efficacy or effectiveness of an intervention by randomly dividing trial participants into experimental or control treatment arms, thereby providing a fair comparison for the unbiased assessment of treatment effects [1-4]. Traditionally, trials have predominantly been conducted in a clinic setting; however, with the increase of the internet as a mainstream communication channel, there has been an increase in the use of email, SMS, and social media for the communication and delivery of interventions [5,6].

### Web-Based Interventions

We defined a web-based or a web intervention as “downloadable or accessible via the internet through a web browser,” which can take the form of (but not limited to) a website, an email, or a web message board. There are various definitions of web-based interventions, some of which include social media and mobile phone apps; however, for the purposes of our review (in particular, our interest in assessing web usage data), we were interested in confining our search to studies that would have been able to assess usage, which until recently was not easy with social media or phone apps. As such, we restricted our definition of web-based interventions accordingly; however, our chosen definition is very similar to that provided by Barak [7].

With an estimated 4.4 billion people being active internet users as of April 2019 [8], an increasing proportion of the global population are potential users of web-based interventions, particularly given the convenience and flexibility of such interventions. As such, these interventions have enormous potential to improve health and health care delivery and can be easily accessible to patients [1,9-11].

### Monitoring Web Usage

In the same way that drug treatments may be prescribed at a certain dose, trial participants receiving a web-based intervention may be advised to use the intervention to a specified degree (eg, in terms of duration or frequency of intervention use). If it is of interest to determine whether trial participants adhered to the recommended intervention dose, it is important to be able to track participants’ intervention use. There are multiple published reviews relating to web-based intervention usage. For example, Kelders et al [12] reviewed the literature to investigate whether study design predicts adherence to a web-based intervention, whereas Perski et al [13] reviewed the literature on digital behavior change interventions to identify or develop a framework linking direct and indirect influences on engagement and the relationship between engagement and intervention effectiveness.

There are numerous automated tools that can be used to track and record a participant’s web intervention use [14]. These tools can be split into two categories, either client (browser) based or server based. Client-based tools, such as Google Analytics (GA) [15], rely on the web browser supporting them (eg, JavaScript being enabled) [16], whereas server-based tools, such as web server log data [17], will always be populated, as they record what data are sent to the client. These tools provide information about participants’ web intervention use, such as which web pages a participant has visited and when a web page has been accessed. However, the reliability of these tools is not guaranteed. Some tools that have been adopted by researchers to measure web usage, such as GA, were not originally designed for accurate reporting of web usage but were instead developed as a marketing aid. As such, while being easily accessible and commonly used, GA may not be the most appropriate tool to use in scientific research [18]. For example, prior research by OBrien et al [19] has demonstrated that 58% of activity on a website is unreported by GA.

To link intervention usage to a particular participant, rather than just obtaining general information about overall intervention use by all participants, each participant requires a unique identifier (UID), such as the study randomization number or a username [20]. The use of a UID facilitates statistical analyses by linking intervention use with outcome data on an individual participant basis. Such data can then be used to inform statistical analysis to estimate the efficacy of the intervention received, rather than simply estimate the effectiveness of the intervention as randomized (as estimated by an intention-to-treat analysis). Commonly used methods to estimate efficacy, using participants’ usage of the assigned intervention, include an as-treated, per-protocol analysis and completer analyses [21]. However, the use of these methods when a trial is subject to deviations from randomized treatment may introduce bias, and more appropriate causal methods should be used, such as complier average causal effect (CACE) analysis [22,23].

### Consolidated Standards of Reporting Trials and Consolidated Standards of Reporting Trials of Electronic and Mobile Health Applications and Online TeleHealth Guidelines

The Consolidated Standards of Reporting Trials (CONSORT) [24] guidelines were introduced in 1996 to improve the consistency and quality of reporting in RCTs. To address the specific challenges of web-based and mobile app–based intervention studies, the Consolidated Standards of Reporting Trials of Electronic and Mobile Health Applications and online TeleHealth (CONSORT-EHEALTH) extension was published in 2011 [25]. This extension encourages trialists to report on participants’ intervention use; subitem 6a-ii of the CONSORT-EHEALTH extension states that researchers should “explain how use and engagement was measured and defined” and subitem 17-I states that “use and usage outcomes should be reported”. The intended benefit of these guidelines will, however, only be realized if they are adhered to; as such, it is important to assess their uptake in trials that have been published since their release.

### Aims and Objectives

This systematic review was conducted to ascertain the extent and nature of web-based intervention use in trials and the current practice among trialists in terms of collecting, reporting, and analyzing web usage data. We were also interested in determining the characteristics of such trials, including the types of design, intervention formats, and clinical areas.

## Methods

### Literature Search

An initial systematic search of PubMed was conducted to ascertain whether there had already been any comprehensive systematic reviews of web-based intervention trials published to date (see Multimedia Appendix 1 for search terms) [12,25] The electronic database, PubMed [26], was then searched to identify all web-based intervention trials published by the end of 2017 (see Textbox 1 for search terms). The protocol for this review has been published in the International Prospective Register of Systematic Reviews [27].

**Search terms for published Web-based intervention trials.**

(online[tiab] OR digital[tiab] OR web-based OR web) AND internet[majr] AND (“Randomized Controlled Trial” [Publication Type] OR randomized control trial OR randomised control trial OR controlled trial OR controlled clinical trial OR RCT) (PLUS manual entry of upper limit of 31/12/2017 for date published)

### Eligibility Screening

Following the removal of duplicate records, all remaining abstracts identified through the PubMed search were screened by an author (EK) to assess eligibility. Only RCTs involving a web-based intervention and published by the end of 2017 were eligible. Studies were excluded if they did not involve a web-based health intervention (eg, educational studies) or were nonrandomized (eg, feasibility studies that did not involve randomization, observational studies, quasi-randomized studies, and surveys), secondary analyses, trial protocols, or systematic reviews. Where there was any uncertainty regarding eligibility, authors DA and SD were consulted, and any disagreements were resolved by consensus. Five percent (77/1540) of the abstracts were randomly selected and assessed for eligibility by authors DA and SD to validate this process, on which there was 100% agreement.

### Data Extraction

A total of 100 studies were randomly selected from the cohort of eligible trials identified in this search, with sampling proportional to the annual distribution of publication years across the entire set of eligible studies. The initial data extraction form was piloted on five studies and refined accordingly. The final dataset included the study characteristics, whether a CONSORT flow diagram and CONSORT-EHEALTH checklist were reported, whether treatment protocol deviations (ie, changes to randomized web-based interventions) were reported, the methods used to collect web usage data, and which statistical analysis methods were used to adjust for intervention use.

## Results

### Review of Systematic Reviews of Web-Based Intervention Trials

The PubMed search for systematic reviews of web-based intervention trials identified 271 citations, 123 of which were found to be eligible following a review of titles and abstracts. These systematic reviews covered a wide range of clinical or methodological areas, most commonly health promotion (47/123, 38.2%) and mental health (40/123, 32.5%; see Multimedia Appendix 2). None of these systematic reviews included a comprehensive search of all published web-based health intervention trials.

### Review of Web-Based Intervention Trials

**Figure 1:**
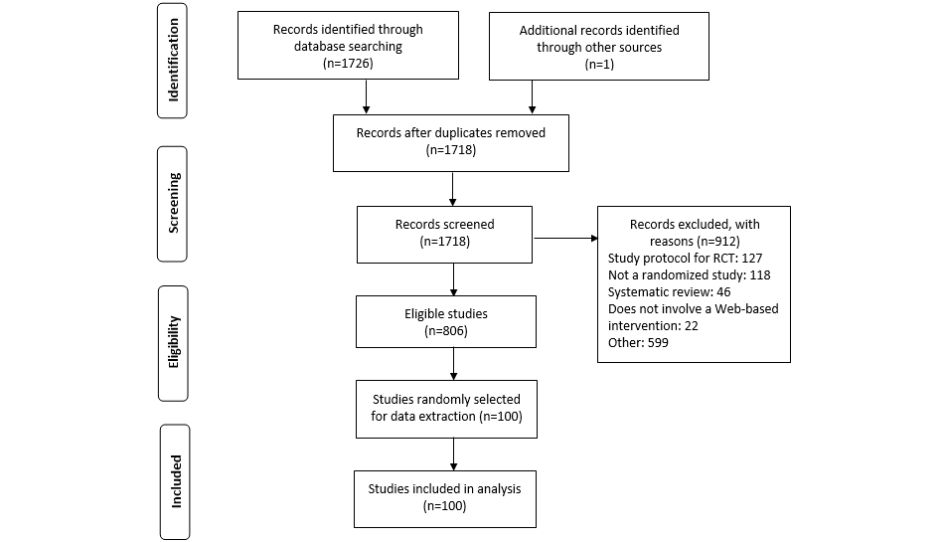
Search and screening process. RCT: randomized controlled trial.

The electronic database search for trials of web-based interventions yielded 1726 publications (Figure 1). After removing nine duplicates, there were 812 eligible and 906 ineligible studies based on the review of abstracts, including one publication identified manually as the original trial report relating to another publication identified in the search. Of the 100 eligible studies selected for data extraction, six were subsequently excluded after reading the full publication. These ineligible studies were replaced with an additional six eligible studies for data extraction.

### Published Web-Based Intervention Trials

**Figure 2:**
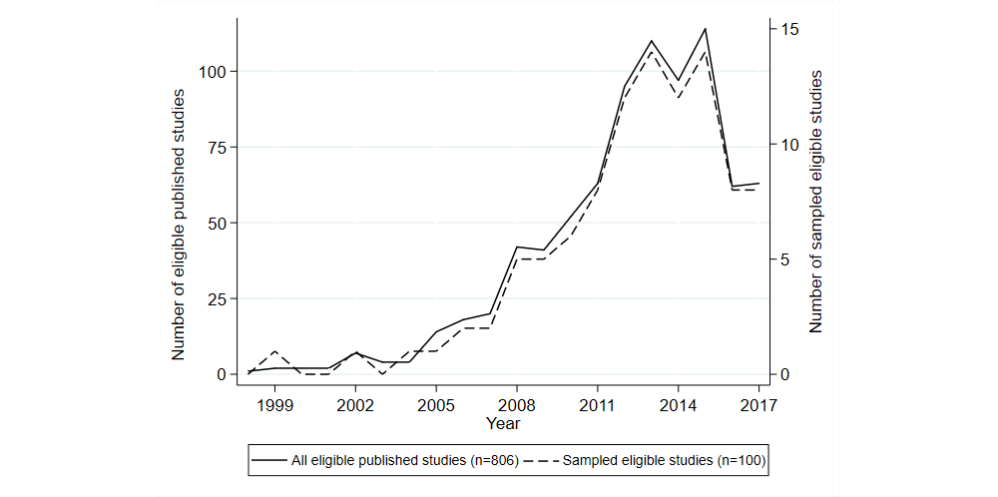
Number of published and sampled trials of online intervention trials each year.

The number of published trials involving web-based interventions is displayed in Figure 2, demonstrating an increasing trend over time. However, despite this increase, the number of trials using web-based interventions remains proportionally low when compared with the total number of trials during this period (estimated as 496,238 from a PubMed search filtered to only include trials published up to the end of 2017). The reduction seen after 2015 is likely to be due to publications not being fully indexed or registered within the PubMed database when the search was run (Feb 12, 2018). A PubMed librarian confirmed that new publications may be posted on PubMed significantly later than their publication date.

### A Description of Studies and Study Characteristics

The characteristics of the 100 publications randomly selected for data extraction are given in Table 1. Most of these studies covered health promotion (42/100, 42.0%; most commonly smoking cessation, physical activity, and weight) and mental health (32/100, 32.0%).

**Table 1:** Characteristics of sampled trials (N=100).

Clinical area		Values
**Health promotion, n**		42
	Smoking cessation	11
	Physical activity	8
	Weight	7
	Alcohol	3
	Eating disorder	3
	Lifestyle behaviors	2
	Physical activity and diet	2
	Diet	2
	Sexual health	1
	Tanning	1
	Adolescent health	1
	General health management	1
Mental health, n		32
Cancer, n		4
Respiratory illnesses, n		3
Neurology, n		3
Diabetes, n		3
Dentistry, n		2
Otolaryngology, n		2
Cardiovascular, n		2
Pain, n		1
Autonomic arousal, n		1
Discharge from emergency department, n		1
Parathyroid disorder, n		1
HIV, n		1
Cancer screening, n		1
Women’s health, n		1
**Design, n**		
	Superiority	94
	Equivalence	4
	Noninferiority	2
**Blinding, n**		
	Double	1
	Single	13
	None	46
	Not stated	40
**Web-based intervention, n**		
	Website	77
	Website plus additional element	10
	Internet (other)	13
**Control arm, n**		
	Website	14
	Internet (other)	14
	Waiting list group	32
	Noninternet intervention	28
	No intervention	9
	Not stated	3
CONSORT^a^ flow diagram presented^b^, n (%)		79 (79.0)
CONSORT-EHEALTH^c^ checklist presented^d^, n (%)		26 (38.2)

^a^CONSORT: Consolidated Standards of Reporting Trials.

^b^The denominator for percentage values is equal to 100, as all trials were published after the original CONSORT flow diagram (1996).

^c^CONSORT-EHEALTH: Consolidated Standards of Reporting Trials of Electronic and Mobile Health Applications and online TeleHealth.

^d^The denominator for percentage values is equal to the number of trials published since the formulation of the CONSORT-EHEALTH (2011; N=68).

The vast majority of trials had a superiority design and did not use blinding or did not state whether there was any blinding. A total of 13 studies reported being single blinded (six reported blinding of the assessors, six reported blinding of the patients, and one reported blinding of the clinician), and only one study reported being double blinded (patients and assessors). In the 86 trials that stated that there was no blinding or did not mention blinding, the web-based and control interventions took different formats—most commonly a website intervention vs a waitlist (n=25) or noninternet (n=18) intervention—which would have made it difficult to blind participants.

The majority of studies involved a website as the intervention; other interventions included a podcast, emails, web applications, a web-based video camera, computer simulation, a computer-generated photoaging intervention, web message boards, an internet partner, a YouTube video, a web-based video, and an internet video conference. In total, 10 studies reported a website plus an additional element, which took the form of a mobile app, a web-based video, social media, an interactive voice response, a personal activity monitor, a personal digital assistant, or an online forum. The most common type of a control arm intervention was waiting list (delayed treatment) followed by noninternet interventions (a face-to-face intervention, written materials, and treatment as usual). Table 2 displays the cross-tabulation of web-based and control interventions in the 100 sampled trials.

**Table 2:** Web-based and control interventions.

Control intervention	Web-based intervention			Total, n
	Website, n	Website plus additional element, n	Internet (other), n	
Website	12	2	0	14
Internet (other)	10	0	4	14
Waiting list group	26	2	4	32
Noninternet intervention	20	4	4	28
No intervention	6	2	1	9
Not stated	3	0	0	3
Total	77	10	13	100

Of the 100 studies, 79 included a CONSORT flow diagram, whereas 38% (26/68 studies published after the CONSORT-EHEALTH guideline, 2011) of studies included a CONSORT-EHEALTH checklist (Table 1).

The publication of CONSORT-EHEALTH does not appear to have positively influenced the rate of reporting web usage (Table 3). 

Of 26 trial publications that included a CONSORT-EHEALTH checklist, four did not report whether web usage data were collected. There were different reasons for not reporting usage in these four publications: one trial acknowledged collecting usage data with the intention to publish usage in a separate publication, one trial did not collect usage because of privacy protection (with no further explanation), one trial gave no explanation on why usage was not collected, and it was not possible to access the CONSORT-EHEALTH checklist in the fourth trial (because of an expired or invalid checklist hyperlink).

**Table 3:** Rates of reporting web usage data according to the publication year and Consolidated Standards of Reporting Trials of Electronic and Mobile Health Applications and online TeleHealth checklist reporting.

Publication year			Reported web usage data		Total
			Yes	No	
≤2011, n (%)			30 (94)	2 (6)	32 (100)
**>2011, n (%)**					
	**Included** **Consolidated Standards of Reporting Trials of Electronic and Mobile Health Applications and online TeleHealth checklist**				
		Yes	22 (85)	4 (15)	26 (100)
		No	38 (91)	4 (10)	42 (100)
Total, n			90	10	100

### Collection and Reporting of Web Usage Data

Commonly used formats for the web-based intervention included sessions (n=17 trials), modules (n=13), content (n=13), and assignments (n=5). Other formats included cartoons, messages, videos, photographs, and various tasks or exercises. Examples of these interventions included a brief personalized normative feedback system provided by various modes of delivery [28], identical content delivered as a podcast or via a website [29], and website information to encourage and support a personalized physical activity plan [30]. One trial [31] used a computer-generated photoaging intervention, with which participants were digitally photoaged and received a photograph of themselves as a lifelong smoker and as a nonsmoker. Exercises took the form of mindfulness exercises as a part of module completion [32] and a series of abdominal plank exercises while exercising with an internet partner [33].

Web usage data were collected in 90 of the studies, but more than half (49/90, 54%) of these studies did not state the method used for recording web usage. The most commonly reported tool used for tracking web usage was a server or electronic log files (see Table 4). Other methods included software tools, website tracking data, GA, and self-reported data. Only 4% (4/90) trial reports mentioned checking the reliability of their web usage measurement methods, two of which used more than two tools to capture and compare web usage data.

**Table 4:** Web usage data collection methods among 90 trials which collected web usage data.

Method and second method (if applicable)		Frequency, n (%^a^)	
Logs		10 (11)	
Software tools		6 (7)	
Website tracking		4 (4)	
**Google Analytics**		5 (6)	
	Alone		3 (3)
	With Logs		2 (2)
**Self-reporting**		5 (6)	
	Alone		3 (3)
	With Logs		1 (1)
	With tracking data		1 (1)
Others		11 (12)	
Not stated		49 (54)	

^a^% of 90 trials which reported web-based intervention use.

Among the 87 trials involving a website, 78 (90%) recorded web usage data, most commonly in terms of the number of log-ins (37/87, 43%), the number of individual intervention components completed (21/87, 24%; eg, assignments, exercises, lessons, and modules), measures of activity on the site (eg, answers entered, activated hyperlinks, blogs, or forum posts; 18/87, 21%), and time spent on the site (18/87, 21%; see Table 5). A total of 36% (31/87) of these trials recorded a combination of two or more usage measures, most commonly the number of log-ins and time spent on the site (15 trials). Among the 23 trials involving a web-based intervention other than a website, 20 (87%) recorded web usage data, most commonly in terms of the number of log-ins (6/23, 26%), video views (6/23, 26%), and measures of activity (5/23, 21%). A total of 26% (6/23) of these trials recorded more than one usage measure (see Table 6). 

**Table 5:** Features of web usage recorded among trials that involved a website (N=87).

Web usage recorded among trials that involved a website	Trials^a^, n (%)
No web usage data collected	9 (10)
Activity on site (eg, answers, activated hyperlinks, and blog or forum posts)	18 (21)
Communication (eg, emails, Skype calls, call logs, and messages sent)	3 (4)
Completed intervention (eg, all assignments, exercises, lessons, or modules)	3 (4)
Number of individual intervention components (eg, modules, sessions) started/accessed	3 (4)
Number of individual intervention components (eg, modules, sessions) completed	21 (24)
Number of log-ins	37 (43)
Number of page hits (individual actions, eg, audio clips, scrolling, and printing)	1 (1)
Number of page views	14 (16)
Time spent on site (including time spent listening to podcast)	18 (21)
Video views (including YouTube views)	1 (1)

^a^Note that 24 trials included two measures of web usage, four trials included three measures of web usage, and three trials included four measures of web usage.

**Table 6:** Features of web usage recorded among trials that involved a web-based intervention other than a website (N=23).

Web usage recorded among trials that involved a website	Trials^a^, n (%)
No web usage data collected	9 (10)
Activity on site (eg, answers, activated hyperlinks, and blog or forum posts)	18 (21)
Communication (eg, emails, Skype calls, call logs, and messages sent)	3 (4)
Completed intervention (eg, all assignments, exercises, lessons, or modules)	3 (4)
Number of individual intervention components (eg, modules, sessions) started/accessed	3 (4)
Number of individual intervention components (eg, modules, sessions) completed	21 (24)
Number of log-ins	37 (43)
Number of page hits (individual actions, eg, audio clips, scrolling, and printing)	1 (1)
Number of page views	14 (16)
Time spent on site (including time spent listening to podcast)	18 (21)
Video views (including YouTube views)	1 (1)

^a^Note that five trials included two measures of web usage, and one trial included three measures of web usage.

A total of 44% (40/90) of trials that collected web usage reported using UIDs, most commonly log-in credentials or internet protocol addresses (see Table 7). An additional 12% (11/90) of publications reported the use of a server or electronic logs to record web usage, both of which have the potential to include UIDs. A total of 8% (7/90) of trials implied having UIDs but did not state what type of UID was used.

**Table 7:** Unique identifiers (N=100).

Unique identifiers	Values, n
Total web usage collected	90
Unique identifier	40
Potential unique identifier (server/electronic logs)	11
Implied unique identifier but not specified	7
No unique identifier	3
Not stated	29

Trialists reported changes to randomized web-based interventions (treatment protocol deviations) in 33 of the studies. Departures from randomized treatment included failing to initiate treatment (in 15 trials, eg, when participants did not activate the account, access the site, or log in); premature discontinuation of the intervention (in 18 trials, eg, when participants withdrew from the trial or experienced difficulties using the site); switching to an alternative arm, which was reported in two trials; and switching to non-web-based treatment, reported in two trials.

### Intervention Dose

A total of 69 trials from our sample specified a recommended dose of the web-based intervention, 62 (90%) of which measured web usage. The dose was specified in terms of sessions, modules, or assignments in 49% (34/69) of these studies (mean 2.8, SD 2.3; range 1-14). Of the 23 studies that reported a time frame for the use of the web-based intervention, the duration ranged from 1 to 12 weeks (mean 2.2, SD 1.3), with the exception of one study, which reported a duration of 150 days (5 months). The average dose frequency was one task per week in 36% (25/69) of studies that recommended a dose. A total of 9% (6/69) of studies reported that participants had more than one task to complete per week, and 10% (7/69) studies reported that participants were due to complete tasks less frequently than 1 per week.

### Analyses Involving web Usage Data

Only 39% (35/90) of trials that collected web usage data investigated the levels of intervention use (Table 8). A total of 21% (19/90) of studies used statistical methods to adjust for intervention usage, such as a completer analysis (11 trials), regression analyses with intervention use as a covariate (six trials), and a CACE analysis (two trials). One of the two trials that used a CACE analysis did not present results or explain their method further, whereas the other trial presented CACE results and explained that the analysis estimates the potential efficacy among participants who would comply with their randomized intervention.

**Table 8:** Analyses involving web intervention use (N=100).

Analyses involving web intervention use	Values, n
Any analysis involving web intervention use	35
Comparison of web intervention use between randomized arms	3
Assessed patterns of web intervention use	4
Correlation between web intervention use and outcome	9
Completer analysis	11
Regression analyses with web intervention use as a covariate	6
Causal analysis (complier average causal effect)	2

## Discussion

### Characteristics of Web-Based Intervention Trials and Systematic Reviews

Although the use of web-based interventions in RCTs has been on the rise over the last 15 years, unsurprisingly, the number is still low in comparison with the overall number of published trials. A random sample of 100 trials suggests that web-based interventions are most commonly used for health promotion (42/100, 42.0%) or mental health issues (32/100, 32.0%), with the remaining 26.0% (26/100) of trials covering 14 clinical areas, including cancer (4/100, 4.0%), diabetes (3/100, 3.0%), and neurology (3/100, 3.0%). The review of systematic reviews of web-based intervention studies demonstrated a similar pattern, with 38.2% (47/123) of reviews relating to health promotion interventions and 32.5% (40/123) relating to mental health. All systematic reviews identified were restricted to trials within a certain clinical condition, other than the review by Mathieu et al [5], which only included trials that were fully or primarily conducted online (eg, involving web-based recruitment, consent, randomization, and follow-up), whereas Lustria et al [34] reviewed trials that defined *electronic health*. As such, this study of systematic reviews demonstrated that there were no previously published reviews of all web-based intervention studies, providing evidence of the novelty and usefulness of this study.

### Adherence to CONSORT and CONSORT-EHEALTH Guidelines

Good quality reporting allows clinicians and researchers to replicate trial methods [35-37] and supports the understanding of trial methods, interventions, and outcomes. This study suggests that there is a need for greater adherence to reporting guidelines in publications of web-based intervention trials. Less than 80% of the trials in our sample presented CONSORT flow diagrams, which is considerably less than the 96% reported to have presented CONSORT flow diagrams in a sample of 100 trials published in 2008 [21]. This may be because of the fact that CONSORT is less commonly endorsed by health informatics journals than clinical journals or is less familiar to trialists assessing web-based interventions than clinical trialists, generally.

Furthermore, although the CONSORT-EHEALTH guideline is listed on the Enhancing the QUAlity and Transparency Of health Research (EQUATOR) website [38] and has been adopted by the Journal of Medical Internet Research, less than 40% of the studies published since CONSORT-EHEALTH was published including a CONSORT-EHEALTH checklist; the authors may, therefore, want to consider some of the strategies suggested by the EQUATOR network to increase the use of guidelines [39], such as further dissemination via journal editorials or conference presentations, the provision of web-based training, or publicity via social media or blog posts. Improving awareness and uptake of the CONSORT-EHEALTH guidelines is important to ensure that the methodological quality of web-based intervention trials is clearly communicated, thereby allowing readers to make informed judgments on the validity of inferences and conclusions drawn in such trials.

### Reporting and Analysis of Web Usage Data

The CONSORT-EHEALTH guideline recommends reporting data collection methods and results relating to intervention use, but not all studies that included a CONSORT-EHEALTH checklist reported information on the collection of web usage data. Indeed, the publication of CONSORT-EHEALTH does not seem to have influenced the quality of reporting regarding web usage, as the rate of reporting web usage data was higher before the publication of CONSORT-EHEALTH.

Unlike drug interventions, the adherence to which can be summarized using uncomplicated measures of treatment intake (eg, initiation, completion, and persistence [21]), web-based interventions often involve multiple features [40,41], engagement with which may be more complex to record. For example, it may be of interest to determine typical navigation patterns through a website, which precise areas of a web page are read or whether videos are watched in their entirety, none of which would be trivial to capture. Our review demonstrated that trialists collect data on a wide variety of web usage features, most commonly the number of log-ins, the number of intervention components completed, activity, and time spent on the site. One-third of the trials that recorded web usage information collected web usage data on more than one feature, the most common combination being the number of log-ins and time spent on the site. The likelihood of measuring web usage data did not vary according to whether or not participants were recommended to follow a specific dose (eg, when participants were asked to use the web-based intervention for a specific period or to complete a certain number of modules): the proportion of trials that measured web usage was equal to 90% in those trials that did (62/69), and in those that did not (28/31), specify a recommended dose. This suggests that the high rate of measuring web usage in web-based intervention trials is not necessarily because of the trialists’ interest in assessing participants’ adherence to a recommended intervention dose; instead, web usage data are commonly recorded regardless of whether there is a recommended dose, demonstrating that such data appear to be of interest to trialists in their own right.

Trialists rarely provided a rationale for their choice of web usage metrics or analysis methods to adjust for web usage. Only two of the 15 trials that adjusted their outcomes for intervention use applied an appropriate method of causal analysis (CACE) to estimate efficacy, suggesting a lack of awareness regarding appropriate methods to account for the impact of participants’ intervention use on their outcomes.

### Assessing the Reliability of Web Usage Data

Although automated capture of participants’ use of web-based interventions may be assumed to be more straightforward and reliable than the usual measures used to capture drug treatment intake (which typically involve participant self-reporting, such as pill counts and treatment diaries, and, therefore, are potentially subject to recall bias or distortion), this is not necessarily the case. Assessing the reliability of web usage data collection methods is, therefore, vital, but very few trialists in our sample mentioned checking the reliability of their web usage measurement methods. When trialists do not check the reliability of their web usage data collection methods, there is a potential for their web usage data (and any subsequent inferences based on these data) to be biased, particularly when inherent features of web usage differ between the randomized interventions. van Rosmalen-Nooijens et al [42] compared the results from GA, content management system logs, and data files with self-reported data from participants and concluded that the usage information from the different sources corresponded well. Nguyen et al [43] and Mermelstein et al [44] also aimed to assess the reliability of their methods, but both studies reported a lack of reliability of their data because of technical or logistical issues. Similar to drug trials, participants’ self-reported web usage may also misrepresent the true use of the intervention [45]. For example, Fleisher et al [46] found discrepancies between self-reported data and usage data obtained from the NetTracker software tool. Fleisher [46] reported that nearly 40% of the participants who reported using the website actually did not log in, whereas 20% of those who reported they did not use the website did, in fact, log in. We are currently undertaking work to determine the reliability of different web usage collection methods, given the uncertainty regarding the accuracy of certain methods.

### Strengths and Limitations

This review was not designed to identify trials that used mobile phone apps or social media interventions. This was a conscious decision because our primary aim was to determine the frequency with which trialists monitored web usage.

A large number of eligible studies prohibited data extraction on all eligible trials; as such, it was decided that a random sample of these trials would be selected (using stratified sampling according to the year of publication to ensure that the publication year profile mirrored that of the complete cohort of eligible studies). Although only 100 of the eligible trial publications were, therefore, included in the data extraction exercise, we believe that this is a sufficient number to give reliable estimates (eg, ensuring the estimation of proportions up to a maximum standard error of 0.05) and an accurate indication of trends in reporting and analysis.

The process of determining the eligibility of web-based intervention trials was based on the review of abstracts only, as such some of the studies deemed as eligible may not have been, as evidenced by the exclusion of six studies from the sample of 100 studies. In addition, only 1 reviewer carried out data extraction; however, this reviewer was able to consult the opinion of a second reviewer if in any doubt so as to appropriate classifications.

This review is limited by the search of only one web-based publication database, PubMed. The number of web-based interventions in 2016/2017 will be underestimated from this search because of delays in registration and indexing of studies within PubMed. PubMed indexes the majority of, but not all, health informatics journals; there are currently 286 health informatics journals, of which 196 are indexed in PubMed. Therefore, a total of 806 trials cannot be taken as the absolute number of web-based intervention trials published up to the end of 2017.

### Conclusions

There is an increasing trend in the use of web-based interventions in RCTs. Tracking web usage data in such trials is necessary to establish the efficacy of web-based interventions. When an intervention is found to be less effective than desired, without usage data, it is hard to determine if the problem is because of the intervention content or the lack of use of the intervention [46]. Information on participants’ intervention use should, therefore, be reported within trial publications with particular focus on relevant features of participation, which are likely to have an impact on outcomes. Although the majority of studies reviewed here reported a measure of web-based intervention usage, trialists often did not report sufficient detail about how the data were collected and rarely considered the accuracy of their web usage data collection methods. There was a modest degree of interest in investigating patterns of web usage, but very few trialists used an appropriate method of analysis to account for the impact of intervention use on participant health outcomes.

## Appendix

Multimedia Appendix 1 Search terms for published systematic reviews of web-based intervention trials.

Multimedia Appendix 2 Clinical or methodological areas covered by systematic reviews identified in search.

## Abbreviations

CACE: complier average causal effect
CONSORT: Consolidated Standards of Reporting Trials
CONSORT-EHEALTH: Consolidated Standards of Reporting Trials of Electronic and Mobile Health Applications and online TeleHealth
EQUATOR: Enhancing the QUAlity and Transparency Of health Research
GA: Google Analytics
RCT: randomized controlled trial
UID: unique identifier
