# Supplementary material for: Usage Metrics of Web-Based Interventions Evaluated in Randomized Controlled Trials: Systematic Review
Source: J Med Internet Res. 2020 Apr 16;22(4):e15474. doi: 10.2196/15474 (PMC7193439; doi:10.2196/15474)
Supplement: Multimedia Appendix 1 [file jmir_v22i4e15474_app1.docx]

| (online[tiab] OR digital[tiab] OR web-based OR web) AND internet[majr] AND  ("Systematic Review"[Publication Type] OR "Systematic Reviews as Topic"[Mesh])  (PLUS manual entry of upper limit of 31/12/2017 for date published) |
| --- |
