# Supplementary material for: Usage Metrics of Web-Based Interventions Evaluated in Randomized Controlled Trials: Systematic Review
Source: J Med Internet Res. 2020 Apr 16;22(4):e15474. doi: 10.2196/15474 (PMC7193439; doi:10.2196/15474)
Supplement: Multimedia Appendix 2 [file jmir_v22i4e15474_app2.docx]

| **Clinical area** |  | **Number (% of 123 systematic reviews)** | |
| --- | --- | --- | --- |
| Breastfeeding |  |  | 1 (0.8) |
| Cancer |  |  | 4 (3.3) |
| Cardiovascular |  |  | 4 (3.3) |
| Caregivers |  |  | 1 (0.8) |
| Chronic health conditions | |  | 1 (0.8) |
| Cyberbullying |  |  | 1 (0.8) |
| Dentistry |  |  | 1 (0.8) |
| Diabetes |  |  | 8 (6.5) |
| HIV |  |  | 3 (2.4) |
| Health promotion^a^ | |  | 47 (38.2) |
|  | Physical activity | 12 (9.8) |  |
|  | Weight | 10 (8.1) |  |
|  | Diet | 10 (8.1) |  |
|  | Alcohol | 7 (5.7) |  |
|  | Smoking cessation | 6 (4.9) |  |
|  | Lifestyle/health behaviours | 6 (4.9) |  |
|  | Sexual health | 3 (2.4) |  |
|  | Gambling | 1 (0.8) |  |
|  | Social | 1 (0.8) |  |
| Insomnia |  |  | 1 (0.8) |
| Health information | |  | 1 (0.8) |
| Networking |  |  | 1 (0.8) |
| Mental Health |  |  | 40 (32.5) |
| Meta-analyses |  |  | 1 (0.8) |
| Neurology |  |  | 1 (0.8) |
| Pain |  |  | 3 (2.4) |
| Phalloplasty |  |  | 1 (0.8) |
| Respiratory |  |  | 1 (0.8) |
| e-trials |  |  | 1 (0.8) |
| e-health definitions | |  | 1 (0.8) |

^a^ Note that 5 reviews covered 2 health promotion areas and 2 reviews covered 3 health promotion areas.
